# Supplementary material for: The comfort in touch: Immediate and lasting effects of handholding on emotional pain
Source: PLoS One. 2021 Feb 9;16(2):e0246753. doi: 10.1371/journal.pone.0246753 (PMC7872251; doi:10.1371/journal.pone.0246753)

Gender Differences

To examine potential gender differences in our outcome variables, we first ran three linear mixed models with gender as the predictor, and emotional pain at first recall, emotional pain during the task, and comfort during the task as outcome variables. There was no significant effect of gender on emotional pain at first recall, *b* = 0.65, *t*(186) = 1.83, *p* = 0.07, 95% CI = [-0.05, 1.35], emotional pain during the task, *b* = 0.35, *t*(184) = 1.21 *p* = 0.23, 95% CI = [-0.22, 0.91], or comfort during the task, *b* = 0.54, *t*(43.63) = 1.02, *p* = 0.31, 95% CI = [-0.49, 1.57].

Next, we included gender as a moderator in our original models including: (i) emotional pain at first recall as a covariate; (ii) valence, touch, gender, and the interactions between them as predictors; and (iii) emotional pain and comfort during the task as separate outcome variables. There was no significant main effect or interaction effects of gender in these models (see Supplementary Table 1-2).

| ***Supplementary Table 1****: Does gender moderate the effects of valence and touch on emotional pain during the task?* | | | | | |
| --- | --- | --- | --- | --- | --- |
|  | *b* | *SE* | *df* | *t* | *p* |
| Valence | -0.86 | 0.33 | 153.96 | -2.61 | 0.01 |
| Touch | 0.17 | 0.29 | 133.68 | 0.59 | 0.55 |
| Gender | 0.47 | 0.31 | 157.51 | 1.48 | 0.14 |
| Emotional Pain Recall 1 | 0.49 | 0.05 | 174.24 | 9.75 | < .001 |
| Valence x Touch | -0.20 | 0.40 | 132.44 | -0.49 | 0.63 |
| Valence x Gender | -0.64 | 0.39 | 132.41 | -1.62 | 0.11 |
| Touch x Gender | -0.44 | 0.40 | 133.04 | -1.10 | 0.27 |
| Valence x Touch x Gender | 0.48 | 0.56 | 132.38 | 0.85 | 0.40 |

| ***Supplementary Table 2****: Does gender moderate the effects of valence and touch on comfort during the task?* | | | | | |
| --- | --- | --- | --- | --- | --- |
|  | *b* | *SE* | *df* | *t* | *p* |
| Valence | 0.34 | 0.70 | 146.53 | 0.49 | 0.62 |
| Touch | 2.96 | 0.60 | 129.63 | 4.96 | 0.00 |
| Gender | 0.68 | 0.72 | 132.77 | 0.95 | 0.35 |
| Emotional Pain Recall 1 | 0.21 | 0.11 | 172.80 | 1.93 | 0.06 |
| Valence x Touch | -1.06 | 0.84 | 128.59 | -1.26 | 0.21 |
| Valence x Gender | -0.53 | 0.83 | 128.56 | -0.64 | 0.53 |
| Touch x Gender | -0.31 | 0.84 | 129.10 | -0.37 | 0.71 |
| Valence x Touch x Gender | 0.43 | 1.18 | 128.53 | 0.37 | 0.71 |

Primary Analyses Using Subset of Participants Who Completed Follow-Up Survey:

Since our follow-up study was completed by a smaller subset of participants (*N* = 31) than the full sample used in our primary analyses (*N* = 47), we re-ran our primary analyses using this smaller subset of participants to ensure consistency in our results. Consistent with our results on the effects of consoling touch on participants’ emotional pain using the full sample, there was a significant main effect of valence, *b* = -0.81, *t*(106.55) = -2.69, *p* = 0.008, 95% CI = [-1.40, -0.23], no main effect of touch, *b* = -0.19, *t*(88.58) = -0.85, *p* = 0.40, 95% CI =[-0.61, 0.24], and no interaction between valence and touch, *b* = 0.23, *t*(88.03) = 0.74, *p* = 0.46, 95% CI =[-0.37, 0.83], on how much emotional pain participants felt, controlling for potential differences in the emotional intensity of the different memories being recalled (i.e. emotional pain at first recall), *b* = 0.61, *t*(116.90) = 9.66, *p* < .001, 95% CI = [0.49, 0.73]. Participants felt significantly more emotional pain during the negative videos (*M* = 4.01, *SD* = 1.75) than the neutral videos (*M* = 1.19, *SD* = 0.26) (Supplementary Figure 1A). Contrary to our hypothesis, pairwise comparisons indicated no significant difference between how much emotional pain participants felt during the consoling touch condition versus the emotional pain only condition, *t*(87.9) = 0.85, *p* = 0.83, 95% CI = [-0.39, 0.77].

Additionally, consistent with our results on the effects of consoling touch on participants’ comfort using the full sample, there was no main effect of valence, *b* = 0.05, *t*(101.56) = 0.08, *p* = 0.94, 95% CI = [-1.28, 1.40], a significant main effect of touch, *b* = 3.12, *t*(87.16) = 6.38, *p* < .001, 95% CI = [2.17, 4.06], and no interaction between valence and touch, *b* = -0.62, *t*(86.71) = -0.90, *p* = 0.37, 95% CI = [-1.96, 0.72], on how comforted participants felt by their partner, controlling for potential differences in the emotional intensity of the different memories being recalled (i.e. emotional pain at first recall), *b* = 0.22, *t*(112.97) = 1.51, *p* = 0.13, 95% CI = [-0.07, 0.51]. Participants felt more comforted by holding their partners’ hand (*M* = 5.45, *SD* = 2.65) than by holding a squeeze ball (*M* = 2.60, *SD* = 2.02) (Supplementary Fig 1B). Consistent with our hypothesis, pairwise comparisons indicated that participants felt significantly more comforted during the consoling touch condition (*M* = 6.13, *SD* = 2.21) than the emotional pain only condition (*M* = 2.94, *SD* = 1.84), *t*(87.7) = -6.38, *p* < .001, 95% CI = [-4.40, -1.84].

**Figure 1**


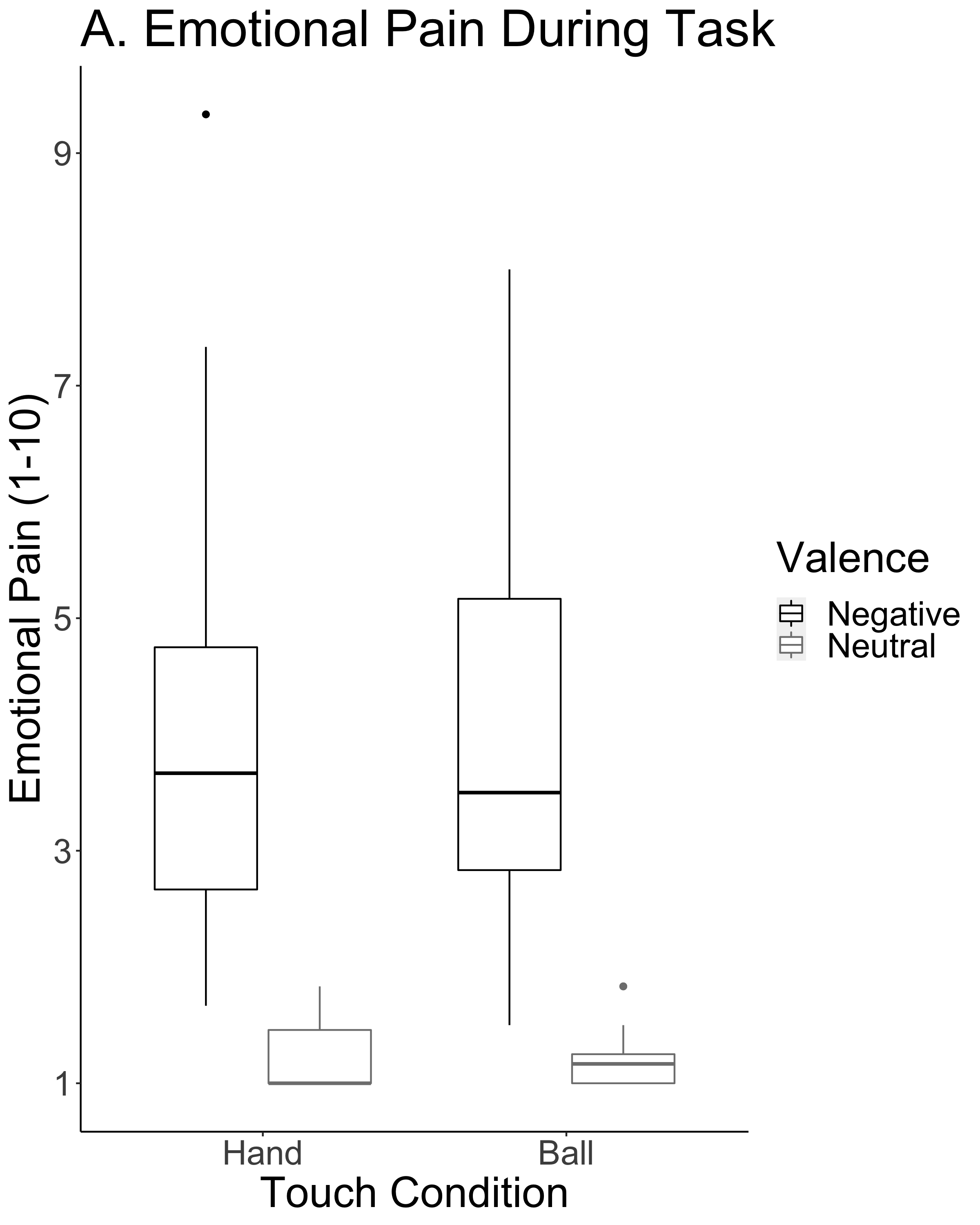

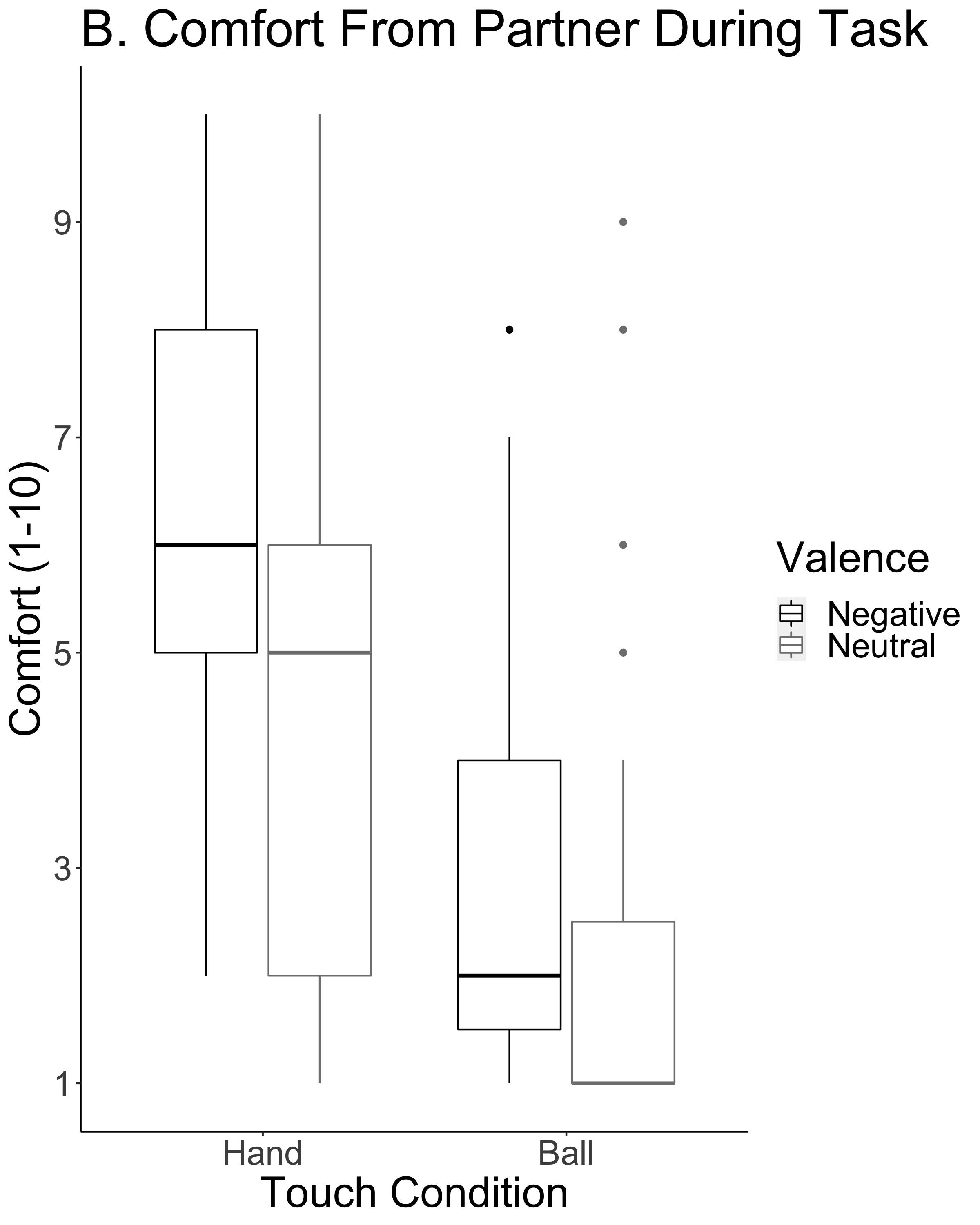

Supplement: S1 File — (DOCX) [file pone.0246753.s001.docx]
