# Supplementary material for: The comfort in touch: Immediate and lasting effects of handholding on emotional pain
Source: PLoS One. 2021 Feb 9;16(2):e0246753. doi: 10.1371/journal.pone.0246753 (PMC7872251; doi:10.1371/journal.pone.0246753)
Supplement: Attachment — Submitted filename: review_plone.docx [file pone.0246753.s002.docx]

-Overview-

This paper, “The Comfort in Touch: Immediate and Lasting Effects of Handholding on Emotional Pain” investigates whether handholding (from a romantic partner) alleviates negative affect associated with the recall of negative life experiences. The authors test for effects in static self-report measures collected both immediately after emotion induction and a few months later. The authors find an effect of handholding on emotional distress in the follow-up period, but not during the task. Handholding did, however, increase feelings of comfort during the task.

The subject of this paper interesting, the paradigm novel, and the results push research on the protective effects of touch forward. The study is well powered and gender balanced (which is rare for touch-analgesia research). I do, however, think it is problematic to rely entirely on subjective self-reports especially when it is noted that NIRs data were collected. Self-report measures are easily influenced by experimental demand characteristics and also do not give us much insight into neuropsychological mechanisms. Furthermore, the stimuli are complex time series data but psychological activity during stimulus presentation is entirely disregarded. These interesting and dynamic stimuli are instead reduced to single self-report values post trial.

I think the paper requires some major revisions, and I expand upon this in detail below.

-Abstract/Introduction-

-Major-

1. This paper is conceptualized as a pain paper, but pain is not studied here. This becomes increasingly problematic as the paper progresses. We are set up to think this paradigm will be nearly identical to those in the pain-domain, however, the stimuli here are high-dimensional personal narrative stories 2-3 min long, whereas pain stimuli tend to be ~7-11 sec stimulations, repeated over many trials with controllable intensity. Then when the results are reported and discussed terms like “emotional pain” and “distress” are sometimes used interchangeably and sometimes used to mean different things. I do appreciate that the authors situated this paper within the context of touch-analgesia research, but I think the introduction does not do justice to the way this paper can converse with that research. I think this happens because negative affect is equated with pain, instead of interrogating both phenomena for what they really are in order to understand the complexity of human experience. I am not suggesting that the connections made with pain research be discarded, but instead, be discussed more critically. For example, some analgesia papers do not just study “pain intensity” but also “pain unpleasantness” which can be considered a more “affective” component of pain than the intensity measure. I think this is a noteworthy bridge to your investigation which explores “unpleasantness” so to speak, but not within any context of physical pain.

Related but more specific requests:

A. Remove the term “emotional pain” from this study entirely. It is not useful to call it “pain” here, it just confuses it with physical pain. I think “distress” is a good term to use throughout. Please operationalize this at the start, and use it consistently (this is particularly confusing in Figures 1 and 3 where one talks about ‘distress’ and the other ‘emotional pain’ but these seems to be the same if not highly correlated measures).

B. At the end of the introduction revisit this sentence: “Given that physical pain is often temporally bound (i.e. restricted to a certain amount of time), whereas emotional pain is often more enduring, it is possible that physical and emotional pain differ in terms of how consoling touch shapes their immediate and lasting experience.”

I think this is a great point, but the way this is written it sounds like it is the *only* difference between them. There are other important differences relevant to this study that can help us to better understand the mechanism supporting touch analgesia. For example, physical pain requires nociceptive input (except in the case of chronic pain). Touch could mediate effects in the periphery or could instead mediate effects at a psychological level. Your study excludes the periphery and therefore could be a nice new piece of evidence to put into conversation with the existing literature.

C. On page 4 “A body of research suggests that physical pain and emotional pain share a common neural system (Eisenberger & Lieberman, 2004, 2005; Eisenberger, Lieberman, & Williams, 2003).” The degree to which these two neural processes are the same this is hotly debated, and it is necessary to acknowledge this debate with appropriate citations for the opposing camp (i.e., Wager et al and all the letters involved in the “Pain in the ACC?” debate) if you do want to discuss this work.

-Minor-

1. Refrain from the term “consoling touch” when describing this investigation — call it what it is — handholding, but link this to the wider concept of consoling touch as appropriate.

3. “This work suggests that handholding, especially with a romantic partner, attenuates subjective distress associated with physical pain, as well as activation in neural regions associated with threat responses (Coan et al., 2013; Coan et al., 2006; Johnson et al., 2013), with some work suggesting that handholding is more effective than other forms of touch, such as gentle stroking, in reducing subjective pain (Reddan, Young, Falkner, López-Solà, & Wager, 2020).” I would be a little careful with the last claim because that Reddan (2020) paper did not find significant differences between the touch conditions on self-reported pain intensity.

4. Consider adding evidence from other neuroimaging papers in addition to the Kraus (2019) to give a more complete survey of what we know about handholding analgesia. The Reddan (2020) paper in particular agrees with your hypothesis about safety conceptualization you put forth in the discussion. There is also:

López-Solà, M., Geuter, S., Koban, L., Coan, J.A., Wager, T.D. (2019). Brain mechanisms of social touch-induced analgesia. *Pain*. 160(9), 2072–2085.

-Method-

-Major-

1. Personal narratives have a lot of variation both within a single story and then also across participants. Emotional content and intensity will fluctuate and sometimes people add a “silver lining” or what they learned from a trying event at the end of their recollection which then can make the story somewhat “positive.” In short, these stimuli are complex! But this complexity is not controlled for or even assessed in any meaningful way. I appreciate that the authors attempted to control for “emotional intensity” in their stimuli via a “memory-related distress” score collected during visit 1, however, I am not convinced this control is valid.

A. Being that the “memory-related distress” score and the “task-related distress” score are the same questions asked of the same people about the same stimuli, I expect these to be highly correlated. Are they? Please test for multicollinearity in all the models which you use the “memory-related distress” score as a nuisance regressor (or the measure where they “recalled how they felt at the time of the event”). If it is correlated with your regressor of interest then it is possible your reported tests are invalid (and this might not be a bad thing — maybe your null findings are actually detectable).

B. Can you attempt to provide some kind of validity check across the stimuli alone? You can, for example, get independent raters to rate the videos moment-by-moment. They you can see if these stimuli are balanced across participants. You can look for the amount of negativity vs like “silver lining” in the negative stories and test if there are outlier stories, etc. This might require you collecting more data on these videos, and I know that can be a big and annoying ask, but I think it is important and won’t be wasted. You can use those “normative” ratings in future studies, etc. Also, if you know these stimuli are balanced you can eliminate the use of that “memory-related distress” nuisance regressor which might be watering down your effects.

2. Was the follow up survey a post hoc addition or part of the original study design? Please specify both at the time when it is first introduced and when it is described in the methods. Address the length of time/gap as well, earlier on.

-Minor-

1. Can you clarify whether the supportive partner could hear or see the story/stimulus beyond the curtain and importantly whether the support receiver was aware of this (could the support receiver possibly think “We are watching this/experiencing this together?”)
2. “The final sample included predominantly Caucasian (39%) and Asian/Asian American (34%) participants (mean age = 21.75 years). “ Please give the complete racial breakdown.
3. Can you clarify if you measured gender identity or biological sex?
4. make clear if the scales were Likert or VAS (It seems like Likert).
5. The term “memory-related distress” is confusing and not ideal.
6. For clarity, please use a different shorthand for your conditions - not “touch-pain condition” etc., to indicate this paradigm is about emotional distress and not physical pain.

-Results-

-Minor-

1. Please include a results table for the non-significant GLMM gender test in your supplementary

2. Please show the data distribution/individual data points in you bar plots

3. Report complete stats for the following two post hoc tests that only report p-values “when additionally controlling for the amount of time that passed between the in-lab manipulation and the completion of the follow-up survey, this effect of touch on current emotional pain did not change (p = 0.03). Relationship satisfaction was not a significant moderator of this effect (p = 0.67). “

-Discussion-

-Minor-

1. There is a history of gender effects in the touch-pain studies you mention (i.e., Coan and Lopez-Sola studies use ONLY female samples, Reddan found gender effects, etc). You found no effects of gender. Please consider discussing this (can be brief).
2. Please reword the following sentence, it is confusing: “Indeed, research suggests that individuals are often motivated to experience certain emotional states even if they feel hedonically unpleasant, because they are in some way instrumental to feel.”
3. Please reword “In other words, we may need to feel certain emotions in order to process and overcome them. “Maybe make this sentence more concrete. I am not sure emotions are something to be “overcome” but maybe bad beliefs or persistent memories underlying the emotions are?

-Misc-

1. Does your IRB require that you restrict data sharing (as described on pg 5 of the compiled manuscript in the "where they data may be found" section)? If not, consider changing this so you can share the data for this or future projects.
2. Please considering including the data that was used to make the composite scores in the uploaded/shareable data.
3. When you used Google Hangouts to monitor your participants, were you able to do this in a way that prevented data-sharing with Google? I haven’t seen Hangouts used like this before, and this is just one of those things that makes me think about how our research practices have to evolve alongside tech/data-mining companies.
4. The mention of the NIRs data being saved for a different study creates concern about dual publication.
