# Supplementary material for: The comfort in touch: Immediate and lasting effects of handholding on emotional pain
Source: PLoS One. 2021 Feb 9;16(2):e0246753. doi: 10.1371/journal.pone.0246753 (PMC7872251; doi:10.1371/journal.pone.0246753)
Supplement: Attachment — Submitted filename: Response to Reviewers.docx [file pone.0246753.s004.docx]

**Round 2 Response to Editor and Reviewers (PONE-D-20-25552R1)**

Editor Comments

**Comment 1:** Thank you for addressing the reviewer's suggestions and revising your manuscript thoroughly. I appreciate your work and would only draw your attention to some minor suggestions by reviewers 1 and 2, which could be addressed, I think.

**Response 1:** We thank you and the reviewers for your second round of feedback to improve upon our manuscript. We have responded to the remaining minor comments in what follows. We hope you’ll agree that this revised manuscript is now ready to share with the scientific community.

Reviewer #1:

**Comment 1:** The authors were appropriately responsive to my comments and have addressed my concerns within the manuscript. I think this will make a nice addition to the literature.
The only lingering comment I would like to make is regarding the word choice of "relationship" between variables of interest. The one change you already made in the in the revised paper reads cleaner and I would suggest updating the other areas of occurrence. Although this is a very minor comment, I think it helps with clarity. Especially as you have a primary variable of interest as "relationship satisfaction" and these terms are used frequently together. See examples of problematic areas below.
p. 18 "... or the relationship between valence and comfort, b =
0.00, t(128.23) = 0.16, p = 0.88, 95% CI = [-0.04, 0.05], but did significantly moderate
the relationship between touch and comfort, b = 0.07, t(128.27) = 2.96, p = .004, 95% CI
= [0.02, 0.11]."
Additionally p. 15 "... Both of these models (assessing the effect of consoling touch on participants’
emotional pain and comfort) were then re-run with participants’ relationship satisfaction
(i.e. CSI) as a possible moderator of the relationship between consoling touch and
emotional pain/comfort. These models included valence, touch, relationship satisfaction,
and interactions between them as predictors, and emotional pain at first recall as a
covariate."

**Response 1:** We thank you for your feedback overall in improving our manuscript, and for this final note to improve the clarity of our results section. We agree that this change helps these sections read cleaner. The recommended changes have been made:

p. 15: “Both of these models (assessing the effect of consoling touch on participants’ emotional pain and comfort) were then re-run with participants’ relationship satisfaction (i.e. CSI) as a possible moderator of the **association** between consoling touch and emotional pain/comfort.”

p. 18: “When including participants’ relationship satisfaction scores in the model as a potential moderator, we found that relationship satisfaction did not moderate the association between valence by touch and comfort, *b* = -0.05, *t*(128.22) = -1.42, *p* = 0.16, 95% CI = [-0.11, 0.02], or the **association** between valence and comfort, *b* = 0.00, *t*(128.23) = 0.16, *p* = 0.88, 95% CI = [-0.04, 0.05], but did significantly moderate the **association** between touch and comfort, *b* = 0.07, *t*(128.27) = 2.96, *p* = .004, 95% CI = [0.02, 0.11].”

Reviewer #2:

**Comment 1:** Thank you for your response and revisions. The authors did not fully address some of my original comments that I think are important for the revision. Below they are listed in order of importance. In your response to comment 12, you did not clarify whether the follow up survey was a post hoc addition or part of the original design. It is critical for the reader to know whether this test was planned a priori or whether it was added on after no effects were found in the main study. It is unclear to me what is meant by exploratory in this context, and this needs to be clarified as this is the finding the entire paper hinges on.

**Response 1:** Thank you for your suggestions to further improve upon our manuscript. We appreciate your point about transparency and have made the nature of this follow-up study more clear on p. 11:

“These surveys were sent out electronically after we completed in-lab data collection for all of our dyads. Thus, participants completed the survey between 1.28 and 7.82 months after session 2 (*M* = 4.01 months). **Because we decided to add this follow-up assessment to our investigation while data collection was ongoing, this portion of the project was an exploratory addition to the original research plan.”**

**Comment 2:** That being said, can you redo the analysis/figures from Figures 1A & 1B (During Task) with just the participants that are included in 3 (Emotional Pain at Follow-up) and include this figure and stats in the supplementary? It would be useful to know if there is an effect for hand v ball "during task" in the reduced sample size which shows an effect "at follow-up."

**Response 2:** We appreciate your point about re-running our analyses with the smaller subset of participants from the follow-up study to ensure consistency with our results using the full sample. We have included detailed information about this in the supplement, and referenced it in the manuscript on p. 16:

“Finally, since this follow-up study involved a smaller subset of participants than our primary analyses, we re-ran our primary analyses using this smaller subset of participants to ensure consistency in our results (see Supplementary Materials for a full report of these analyses and accompanying figures).”

Our results from the primary analyses using the full dataset (*N* = 47) and the reduced dataset (*N* = 31) are consistent (see p. 2-3 of Supplement):

“Since our follow-up study was completed by a smaller subset of participants (*N* = 31) than the full sample used in our primary analyses (*N* = 47), we re-ran our primary analyses using this smaller subset of participants to ensure consistency in our results. Consistent with our results on the effects of consoling touch on participants’ emotional pain using the full sample, there was a significant main effect of valence, *b* = -0.81, *t*(106.55) = -2.69, *p* = 0.008, 95% CI = [-1.40, -0.23], no main effect of touch, *b* = -0.19, *t*(88.58) = -0.85, *p* = 0.40, 95% CI =[-0.61, 0.24], and no interaction between valence and touch, *b* = 0.23, *t*(88.03) = 0.74, *p* = 0.46, 95% CI =[-0.37, 0.83], on how much emotional pain participants felt, controlling for potential differences in the emotional intensity of the different memories being recalled (i.e. emotional pain at first recall), *b* = 0.61, *t*(116.90) = 9.66, *p* < .001, 95% CI = [0.49, 0.73]. Participants felt significantly more emotional pain during the negative videos (*M* = 4.01, *SD* = 1.75) than the neutral videos (*M* = 1.19, *SD* = 0.26) (Supplementary Figure 1A). Contrary to our hypothesis, pairwise comparisons indicated no significant difference between how much emotional pain participants felt during the consoling touch condition versus the emotional pain only condition, *t*(87.9) = 0.85, *p* = 0.83, 95% CI = [-0.39, 0.77].

Additionally, consistent with our results on the effects of consoling touch on participants’ comfort using the full sample, there was no main effect of valence, *b* = 0.05, *t*(101.56) = 0.08, *p* = 0.94, 95% CI = [-1.28, 1.40], a significant main effect of touch, *b* = 3.12, *t*(87.16) = 6.38, *p* < .001, 95% CI = [2.17, 4.06], and no interaction between valence and touch, *b* = -0.62, *t*(86.71) = -0.90, *p* = 0.37, 95% CI = [-1.96, 0.72], on how comforted participants felt by their partner, controlling for potential differences in the emotional intensity of the different memories being recalled (i.e. emotional pain at first recall), *b* = 0.22, *t*(112.97) = 1.51, *p* = 0.13, 95% CI = [-0.07, 0.51]. Participants felt more comforted by holding their partners’ hand (*M* = 5.45, *SD* = 2.65) than by holding a squeeze ball (*M* = 2.60, *SD* = 2.02) (Supplementary Fig 1B). Consistent with our hypothesis, pairwise comparisons indicated that participants felt significantly more comforted during the consoling touch condition *M* = 6.13, *SD* = 2.21) than the emotional pain only condition (*M* = 2.94, *SD* = 1.84), *t*(87.7) = -6.38, *p* < .001, 95% CI = [-4.40, -1.84].”

**Comment 3:** I like the authors' new addition to the discussion on pg 4 (“While physical pain and emotional pain differ….” etc.), however, I think the preceding discussion of the common neural systems (“A body of research suggests that physical pain and emotional pain share a common neural system [22–24], although the extent of this overlap is still debated. …. Additionally,”) should be removed or revised again for the following reasons:
(a) The authors did not add any neural or physiological data to their revision, so bringing up this neuroimaging debate is out of place.
(b) The affective components of physical pain are not equivalent to what is being described as “emotional pain” here, so the beginning of this paragraph makes the new addition (which is more pertinent) confusing.
(c) there is an effect of handholding (v squeeze ball) on self-reports of physical pain “during task” that has been replicated-- there is no effect “during task” here. This is not addressed.
(d) Finally, claims like “share a common neural system” are somewhat meaningless since that common neural system is the brain, and “overlap” does not provide evidence of shared regulatory processes.

**Response 3:** We appreciate your suggestions to streamline our introduction for relavance and have removed the majority of the discussion of the debate on the neural underpinnings of physical versus emotional pain on p. 4 as you now recommend. However, we have modified and retained one sentence to reference the literature on shared neural regions, as well as a reference to the debate on this topic, because we do think it is important to point out that there has been considerable research on this topic, and this neuroimaging research has served as the inspiration and rationale for the current research project. Just as data from animal research can inspire research in humans, and physiological data can inspire a reaction time study, neuroimaging research can inspire a behavioral study. Thus, this neuroimaging research is relavant to our present research question and we believe it should be mentioned in the introduction. Please see the changes made below on p. 4:

“A body of research suggests that physical pain and emotional pain **are processed in overlapping neural regions** [22–24], **although the extent of this overlap is still debated [25].** ~~Specifically, while physical pain versus no physical pain, and emotional pain (i.e. social rejection) versus no emotional pain are differentiated in the same regions of the brain, physical and emotional pain are represented differently in those regions [25]. Nonetheless, such neural overlap between physical and emotional pain suggests that touch may similarly regulate physical and emotional pain. Additionally,~~ While physical pain and emotional pain differ insofar as physical pain has a sensory component (e.g. stinging, burning) [26] and emotional pain stems from psychological events rather than physical stimulation [2], they both involve an affective component (e.g. unpleasantness, distress). Prior research suggests that consoling touch reduces the affective component of physical pain [27], suggesting that consoling touch also has the potential to reduce the subjective unpleasantness associated with emotional pain.”

With regard to point C, that there is no effect of “task” in this study like in studies of physical pain, that is not quite accurate. Studies that examine the effect of handholding on physical pain have two conditions: pain during handholding and pain without handholding. These studies show that self-reported pain is reduced during handholding (vs. no handholding). In the current study, we find that while subjects do not report less emotional pain during the consoling touch condition (emotional pain with touch) as compared to the emotional pain only condition (emotional pain without touch), participants did report higher levels of comfort during the consoling touch condition. Thus, there is an effect of task in the current study, but with a different descriptor (participants express it in terms of more comfort rather than less emotional pain).

Finally, with respect to point D, that claims about “shared neural systems” are meaningless, we respectfully disagree. For example, showing that seeing a stimulus and imagining a stimulus both activate the same region of occipital cortex suggests common processes underlying these two experiences (Kosslyn et al., 1995, Nature). Thus, while this reviewer may not agree that physical and social pain share similar underlying neural circuitry, and this debate is important to reference as we have, we don’t think the idea of shared neural circuitry is meaningless, uninteresting, or unimportant. (Please note that we also changed the language “shared neural system” to “overlapping neural regions” to address any readers who might interpret “shared neural system” as referring to the entire brain; this was not our intended meaning.)

**Comment 4:** To comment 1, the authors responded, “However, please keep in mind that NIRS does not provide access to subcortical neural regions that are informative with regards to the experience of emotional pain, and thus our best measure of emotional pain in this study is from participants’ self-report.”

What was the motivation to collected NIRS data if you did not expect to acquire signal relevant to your study? Would you not expect relevant signal in the PFC, considering its role in emotion regulation? In general, I think my point with regards to an over reliance on self-report data was missed. I was not suggesting that self-report is useless - it is not, it captures one's subject experience. This paper, however, is very limited in its contribution to the literature because it relies entirely on subjective self-report.

**Response 4:** We would like to share more information with you about the NIRS portion of the research to help clarify what we aimed to study, and why we are not sharing this data. We collected NIRS data to begin a preliminary exploration of the neural processes underlying consoling touch during emotional pain to help inform a future fMRI study that we are currently in the process of planning. Specifically, we hyperscanned both participants in each dyad (the person receiving support and the person providing support) to assess whether participants were more “in sync” during consoling touch, similar to Dr. Shamay-Tsoory’s work on inter-partner coupling during physical pain (Goldstein, Weissman-Fogel, & Shamay-Tsoory, 2017). However, we had several issues with data collection that preclude us from reliabily inferring neural activity during the task. Most notably, participant head motion lead the fNIRS caps to slide around on their heads such that the position of the optodes shifted signficantly across the task. Given that we were hyperscanning participants, if even one participant from each dyad had this issue, we were unable to use their data to assess neural synchrony as this motion makes it really difficult to attain spatial specificity in our analyses. As mentioned, we plan to follow-up on this behavioral work with an fMRI study. However, we would like to underscore your point regarding the importance of self-report in capturing participants’ *subjective experience* of emotional pain. While this study produces many questions about what is happening during consoling touch in the context of emotional pain, it is the first step towards understanding this experience. We do not believe the absence of neuroimaging data in this paper strips our findings of any value, and believe this work will be generative for future research in this area, and thus should be shared with the scientific community.

Reviewer #3:

**Comment 1:** I'm fine with the current version of the manuscript. Merry Christmas, Happy Hanuka and Happy New Year!

**Response 1:** Thank you for your helpful feedback! Happy Holidays to you as well!
